# Reproduction dynamics of planktonic microbial eukaryotes in the open ocean

**DOI:** 10.1098/rsif.2021.0860

**Published:** 2022-02-16

**Authors:** Manuel F. G. Weinkauf, Michael Siccha, Agnes K. M. Weiner

**Affiliations:** ^1^ Institute of Geology and Palaeontology, Univerzita Karlova, 128 43 Praha, Czech Republic; ^2^ Department of Earth Sciences, Université de Genève, 1205 Genève, Switzerland; ^3^ Center for Marine Environmental Sciences (MARUM), Universität Bremen, 28359 Bremen, Germany; ^4^ NORCE Climate and Environment, NORCE Norwegian Research Centre AS, 5007 Bergen, Norway

**Keywords:** individual-based model, planktonic eukaryotes, population dynamics, reproduction ecology, sexual versus asexual reproduction, survival capacity‌

## Abstract

Understanding the biology of reproduction of an organismal lineage is important for retracing key evolutionary processes, yet gaining detailed insights often poses major challenges. Planktonic Foraminifera are globally distributed marine microbial eukaryotes and important contributors to the global carbon cycle. They cannot routinely be cultured under laboratory conditions across generations, and thus details of their life cycle remain incomplete. The production of flagellated gametes has long been taken as an indication of exclusively sexual reproduction, but recent research suggests the existence of an additional asexual generation in the life cycle. To gain a better understanding of the reproductive biology of planktonic Foraminifera, we applied a dynamic, individual-based modelling approach with parameters based on laboratory and field observations to test if sexual reproduction is sufficient for maintaining viable populations. We show that temporal synchronization and potentially spatial concentration of gamete release seems inevitable for maintenance of the population under sexual reproduction. We hypothesize that sexual reproduction is likely beneficial during the adaptation to new environments, while population sustenance in stable environments can be ensured through asexual reproduction.

## 1. Introduction

The mode of reproduction exerts a strong influence on the evolution of organisms [1,2] and has ramifications for adaptation, diversification and speciation as well as population dynamics. Sexual reproduction is assumed to be a widespread, ancient trait among eukaryotes, including microbial taxa [3]. It has a major influence on populations since gene flow alters genetic diversity and the potential for local adaptations and speciation.

In the open ocean, exclusively sexually reproducing planktonic organisms are confronted with the problem of mate encounter over wide geographical areas to ensure sufficient reproductive success to maintain viable populations. Nevertheless, several plankton species are cosmopolitan and can maintain gene flow on a global scale [4–6]. Purely sexually reproducing planktonic species must have developed efficient strategies to overcome the obstacle of mate encounter to maintain gene flow across areas with low population densities. Such adaptive strategies include the synchronization of reproduction in time or space [7–9], mate detection mechanisms through chemical communication [10], the generation of reproductive cysts [11], or the production of eggs [12] or numerous motile gametes [13,14]. The micro- and meso-zooplankton, such as marine planktonic protists, are especially affected by this problem: due to their small size and limited means for active locomotion [15], they are subjected to passive dispersal by ocean currents. Knowledge on the reproduction of many protists in their natural habitat remains limited due to intrinsic difficulties of laboratory culturing. Consequently, many protist groups are assumed to reproduce purely asexually [16,17], while alternations of sexual and asexual stages [18,19] and exclusively sexual reproduction have been assumed for others [20].

Planktonic Foraminifera are non-motile marine protists with calcitic shells that make up a sizeable proportion of the meso-zooplankton in the world's oceans [21]. Due to their ubiquitous fossil record, they represent excellent model organisms for studying the evolutionary history of marine protists [22]. However, full exploitation of their potential as model organisms in evolutionary studies is hampered by the fact that our knowledge of their modes of reproduction remains scarce. Culturing experiments revealed the formation of large numbers of biflagellated gametes that were released into the surrounding water [13,23]. The formation of these gametes was taken as an indication for sexual reproduction, although fusion of gametes and zygote formation has been described only once [24]. Asexual reproduction has yet only been detected in rare cases and has been estimated to constitute less than 1% of reproductive events [25]. Although recent papers described events of asexual reproduction in two species of planktonic Foraminifera [26,27], it is still speculative how prevalent asexual reproduction is in this group or what triggers its occurrence. Consequently, planktonic Foraminifera are traditionally assumed to be mainly sexually reproducing, dioecious organisms [23]. This contrasts with benthic Foraminifera, among which a heterophasic life cycle with alternating sexual and asexual generations dominates [20]. Since planktonic species evolved from benthic ancestors [28], it was argued that switching to purely sexual reproduction represents an important step in the evolution from a benthic to a pelagic lifestyle [29]. However, when considering the patchy occurrence of planktonic Foraminifera in the open ocean with very low population densities [30,31], the question arises as to whether viable populations can be maintained by sexual reproduction alone. So far, it remains debated if this can be achieved by random gamete encounters or if further adaptive strategies, like temporal/spatial synchronization of reproduction as suggested for some species [7–9,32] or communication via chemical traits, are necessary to enhance gamete encounter rates.

Here, we supplement observations from field and laboratory experiments with mathematical modelling. Based on common framework parameters, we model the rates of gamete fusion by chance encounter. We further estimate the number of zygotes that must survive and reach a reproductive state to maintain the population. We aim to answer the following questions: (i) Can planktonic Foraminifera maintain their populations in the open ocean by relying on sexual reproduction or does asexual reproduction play a more important role than previously assumed? (ii) How is reproductive success distributed across the population and what does this mean for gene flow within the population? (iii) Under which circumstances is either sexual or asexual reproduction favourable?

## 2. Material and methods

We modelled sexual reproduction of planktonic Foraminifera in MatLab v. R2017b to test possible reproductive strategies in nature. An entirely realistic model, with several hundreds of thousands of particles interacting in a turbulent three-dimensional environment, was computationally not feasible. Our model, therefore, comprises several parametrizations and models planktonic Foraminifera and their gametes in a laminar flow environment.

### 2.1. Choice of framework parameters

The range of the framework parameters used for the models was estimated based on literature values and data. Five parameters and their variation ranges were estimated for use in our models (table 1). We selected parameters for modification based on their most likely explanatory power regarding the benefits or disadvantages of different reproductive strategies.

**Table 1.** Summary of framework parameters used for reproduction modelling of planktonic Foraminifera and the source of this information.

	minimum	maximum	source
experiment duration (h)	48	720	
gamete release synchronization (h)	12	756	literature
Foraminifera density (specimens m^−3^)	10	40	literature
gamete size (µm)	2	4	literature
gamete number (*n*)	12 500	400 000	calculation, this study
gamete speed (µm s^−1^)	25	100	video observation

Gamete release is observed frequently (*ca* 30% of all individuals) under laboratory conditions [13,] but has never been observed in nature. Population analyses with high temporal resolution suggested gametogenesis in planktonic Foraminifera to be synchronized in time, with a lunar or semi-lunar cyclicity as a potential trigger for gamete release [7,8,32,36,37]. Accordingly, for most experiments, we assumed a synchronized gamete release within 12 h. We also explicitly tested the effect of a largely unsynchronized release by modelling scenarios where the gametes were released over a period between 36 and 756 h.

To estimate a realistic range of Foraminifera population densities, we used data from research cruises, which took standardized water sample volumes with plankton net tows [30,31,38–40]. For our models, we assumed 10–40 specimens m^−3^ for planktonic foraminiferal densities for most species in mesotrophic environments.

The size and shape of planktonic foraminiferal gametes was described in detail for *Hastigerina pelagica* as being ‘3–4 µm in diameter’ [33, p. 429]. Other sources assume a gamete size of 3–5 µm [23,24], but observations are rare and restricted to a few species. For our models, we used two conservative size estimates of 2 or 4 µm gamete size.

It is challenging to estimate the total number of gametes that are produced by one adult cell due to a lack of published data. A rough estimate for gamete numbers is given in Bé & Anderson [13], who estimated that *Trilobatus sacculifer* produces at least 2.8 × 10^5^ gametes, but who state that probably considerably more gametes had been produced as many had already escaped the shell at the time of observation. Schiebel & Hemleben [23] estimate the average number of gametes produced by planktonic Foraminifera to be approximately 300 000–400 000.

We further used data from studies of calcification intensity in planktonic Foraminifera [41–45] (electronic supplementary material, S1) to estimate gamete numbers for several species. We used size–weight data of individual foraminiferal shells and the density of calcite (*ρ* = 2.7102 g cm^−3^ [46]), to estimate the volume of cytoplasm within the shells (electronic supplementary material, S1). Using some other assumptions (electronic supplementary material, S2) and a gamete size of 30 µm^3^ (approx. 4 µm in diameter), we estimated the production of few tens of thousands to several hundreds of thousands of gametes per individual, depending on the species (electronic supplementary material, S2). Convincingly, our estimate for *Trilobatus sacculifer* is *ca* 500 000 gametes per individual, which is close to the estimate by Bé & Anderson [13]. Based on these results, we ran our models with a range of 12 500 to 400 000 gametes per individual, which covers the confidence intervals of our estimate range.

To our knowledge, no observations about gamete speed in planktonic Foraminifera have been published in peer-reviewed sources so far. However, we used a video by Jennifer Fehrenbacher (Oregon State University, USA) available on YouTube (https://www.youtube.com/watch?v=iCqcKjeqR4g) to estimate gamete speeds. The video shows a specimen of the species *Neogloboquadrina dutertrei* releasing gametes in a culturing dish. We used the size of the foraminifer of 300 µm to approximate the scale of the image and estimated the distance covered by individual gametes, which appears to be 8–12 gamete body lengths per second. Accounting for estimation errors, we ran our models with a range of 25–100 µm s^−1^ as gamete speeds. The video also shows that gametes are explosively expelled to a distance of *ca* 2000 µm, but this initial expulsion is not characteristic of the gametes' own motion and was thus not included in our assumptions.

### 2.2. Model description

**Figure 1.**
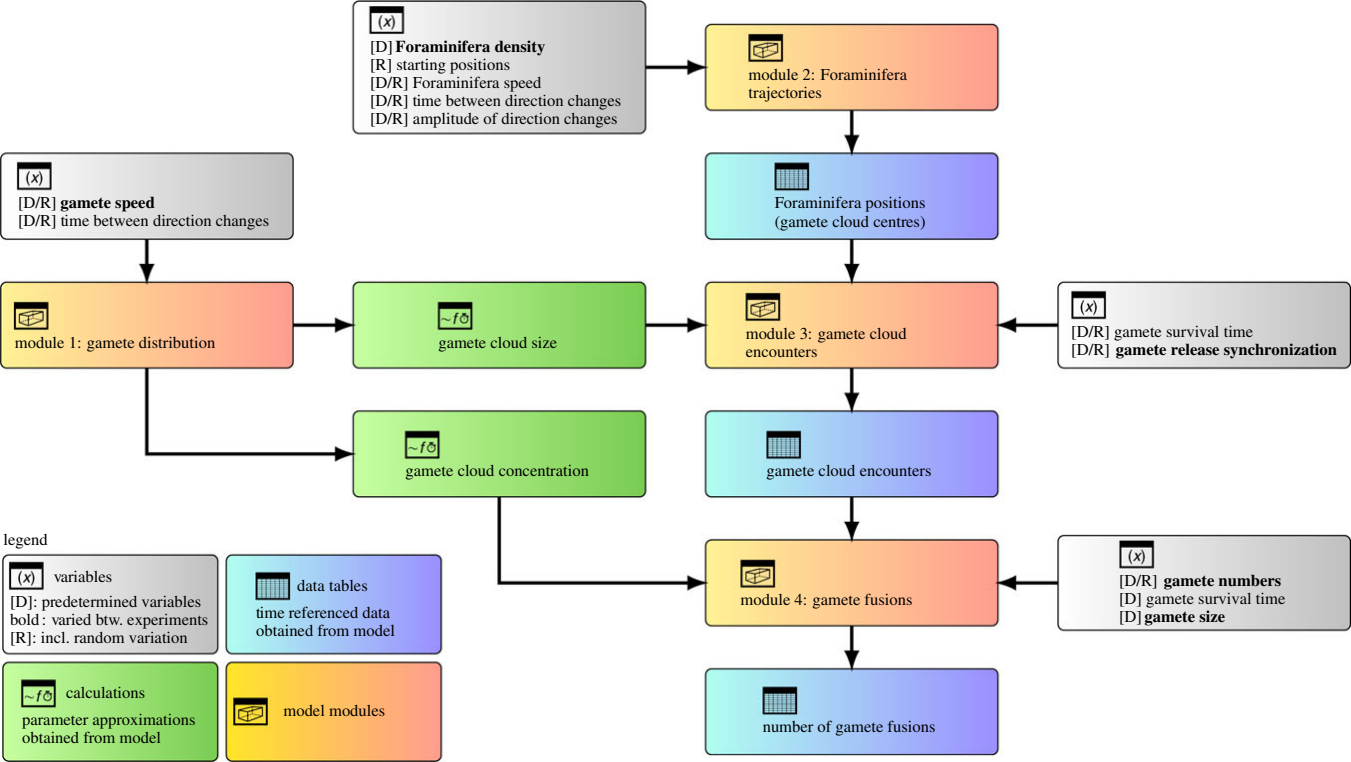
Schematic depiction of the Foraminifer reproduction model designed for this study.

We used a modular model design, which allowed the reuse of experimental set-ups so that for example the trajectories and spawning parameters generated for one experiment could be analysed with varying numbers of released gametes. The following module descriptions are summaries and the model is schematically depicted in figure 1; more detailed descriptions of all modules are available in the electronic supplementary material, S2.

Module 1 simulated the distribution of gametes by a random walk process. Gamete release was simulated with 10 000 dimensionless particles at the centre of a virtual volume which moved along a uniformly randomized vector with regular direction changes. Gamete densities as a function of distance from the centre were approximated by a normal distribution. The fit of this approximation was very good (root-mean-square error: 0.07–0.30%). For later use in Module 3, the maximum distance travelled against time was approximated by a second-degree polynomial function.

Module 2 simulated the movement of adult Foraminifera in a cubic metre of sea water in a quasi-laminar flow. Starting at random initial positions, Foraminifera were moved along a randomly determined fixed starting vector which was altered at random. The purpose of this module was the incorporation of another random element for gamete cloud interaction. Like in Module 1, the individual Foraminifera were dimensionless and could not interact.

Module 3 determined the probability of encounters of the gamete clouds of different adults based on the output from Modules 1 and 2 (we disregard autogamy as an option, as it has never been observed to occur in culture). All adult cells released their gametes within the synchronization time of the respective experiment. The number of released gametes was generated as a normally distributed random number with a mean according to the settings of the experiment (table 1) and a standard deviation of 20%. The maximum distance of travel for the gametes was taken from Module 1 results. The list of probable gamete interaction events was stored for final analysis in Module 4.

Module 4 determined the number of gamete fusions into zygotes. Since our simulated gametes perform a random walk, we used the equation for particle collisions from kinetic gas theory (equation (2.1)):

2.1 $$z=\pi \times d_{G}^{2}\times v_{G}\times n_{A}\times n_{B},$$

where *z* is the collision frequency, *d_G_* is the gamete diameter, *v_G_* is the gamete velocity and *n_A_* and *n_B_* are the concentrations of the gametes of Foraminifera individuals *A* and *B*.

The validity of this approximation was tested by comparing the collision rates obtained from equation (2.1) with direct numerical simulations of the upper triangular matrix of gamete concentrations in the set [5..100,5] gametes ml^−3^.

### 2.3. Data analysis

All model results were analysed in R v. 4.0.2 [47]. The relationship between model parameters and reproductive success was modelled using generalized additive models (GAMs) of the form presented in equation (2.2) as implemented in the R-package ‘gamlss’ v. 5.1-7 [48], using their own fitting algorithm. Dependent variables in the GAMs were fitted via maximum likelihood with *P*-splines based on singular value decomposition. The family of the link-function for the GAMs was chosen based on an evaluation of the distribution of the dependent variable in the R-package ‘fitdistrplus’ v. 1.1-1 [49].

2.2 $$RS=\ell (t_{S})+\ell (n_{F})+\ell (v_{G})+\ell (d_{G})+\ell (n_{G})+\varepsilon ,$$

where *RS* is the reproductive success parameter to be modelled, *t_S_* is the degree of synchronization (i.e. time-window size during which gametes are released), *n_F_* is the density of Foraminifera, *v_G_* is the gamete velocity, *d_G_* is the gamete diameter, *n_G_* is the number of gametes released per foraminifer, *ε* is the error term and *ℓ* denotes a *P*-spline smooth.

The variation of reproductive success parameters was calculated as coefficient of variation CV = *s*/*µ*, where *s* is the standard deviation and *µ* is the mean of the parameter. To estimate the distribution pattern of reproductive success across individuals (i.e. whether all individuals had the same reproductive success), we used Ripley's *L* function [50] and visualized influential parameters across the space of largest variation using flexible discriminant analysis (FDA) [51] as implemented in the R-package ‘mda’ v. 0.5-2. The survival rate of planktonic Foraminifera has been estimated using nonlinear regression.

## 3. Results

Different experimental set-ups strongly varied in their individual reproductive success and are summarized in table 2 and electronic supplementary material, S2. On average, 5.3 individual Foraminifera were able to successfully reproduce per experimental run, generating a total of 604.3 zygotes. With the generated data, we evaluated reproductive success in two ways. (i) We estimated the number of zygotes produced (i.e. successful fusions of two gametes) per successful individual and in relation to the population. These numbers are critical, as sufficient zygotes are required to sustain the population. (ii) We estimated the proportion of the population that was able to successfully reproduce and the distribution of reproductive success across individuals. This is a quantity for the reproductive success among planktonic Foraminifera, which has an impact on population viability and gene flow.

**Table 2.** Summary of results from the planktonic Foraminifera reproduction model. Coeff. var.: coefficient of variation; ind.: individual Foraminifera; pop.: population of planktonic Foraminifera.

parameter	range	mean	3rd quartile
reproducing Foraminifera (*n*)	0–39	5.30	5.00
total zygotes produced (*n*)	0–228 000	604.30	16.35
zygotes per successful ind. (*n*)	0–114 000	94.00	2.00
zygotes per ind. across pop. (*n*)	0–11 400	21.00	1.00
coeff. var. gamete fusion per ind. (*n*, only successful ind.)	0.78–20.37	4.50	5.07
coeff. var. gamete fusion per ind. (*n*, entire pop.)	0.77–20.37	4.35	4.88
proportion of pop. reproducing (%)	0–97.5	17.70	26.70
coeff. var pop.-wide success rate	0.07–10.00	1.54	1.83

### 3.1. Scale of reproductive success

We find that across all experiments, an average of 94 zygotes were produced by each successful foraminifer. Across the entire population, compensating for unsuccessful individuals, this translates to 21 zygotes per foraminifer on average.

The individual success in producing zygotes followed a strongly positively skewed gamma distribution, containing large numbers of zero values. We, therefore, chose a zero-adjusted gamma distribution as link-function for the GAM (results are shown in table 3). We observe that when only considering the successful individuals, synchronization time and Foraminifera density did not significantly influence reproductive success. Gamete speed shows a negative relationship with zygote production, while both gamete size and the number of gametes produced per foraminifer positively influenced the number of fusions (electronic supplementary material, S2). The CV of fusions per individual is 4.50 on average. The variation follows a gamma distribution, and the fitted GAM implies that variation increased with a relaxation of synchronization and decreased with foraminiferal density and gamete speed (electronic supplementary material, S2). Conversely, gamete size and number of gametes produced did not affect the variation of reproductive success.

**Table 3.** Results from a GAM of foraminiferal reproductive success in dependence of model parameters.

	zygotes per successful individual		zygotes across population		successful proportion of population	
	*t*-value	*p*-value	*t*-value	*p*-value	*t*-value	*p*-value
values						
intercept	4.021	<0.001	–3.571	<0.001	−47.888	<0.001
gamete release synchronization (h)	−1.018	0.309	−9.656	<0.001	−59.269	<0.001
foraminifera density (specimens m^−3^)	0.501	0.616	3.230	0.001	41.635	<0.001
gamete size (µm)	6.488	<0.001	6.394	<0.001	−4.162	<0.001
gamete number (*n*)	20.881	<0.001	20.817	<0.001	2.032	0.042
gamete speed (µm s^−1^)	−8.330	<0.001	2.484	0.013	77.086	<0.001
variation of values						
intercept	10.952	<0.001	10.964	<0.001	8.147	<0.001
gamete release synchronization (h)	11.209	<0.001	12.267	<0.001	21.465	<0.001
foraminifera density (specimens m^−3^)	−12.766	<0.001	−12.305	<0.001	−19.930	<0.001
gamete size (µm)	0.883	0.381	1.405	0.166	2.193	0.033
gamete number (*n*)	−0.911	0.366	−0.896	0.374	−1.032	0.307
gamete speed (µm s^−1^)	−9.743	<0.001	−11.188	<0.001	−23.756	<0.001

**Figure 2.**
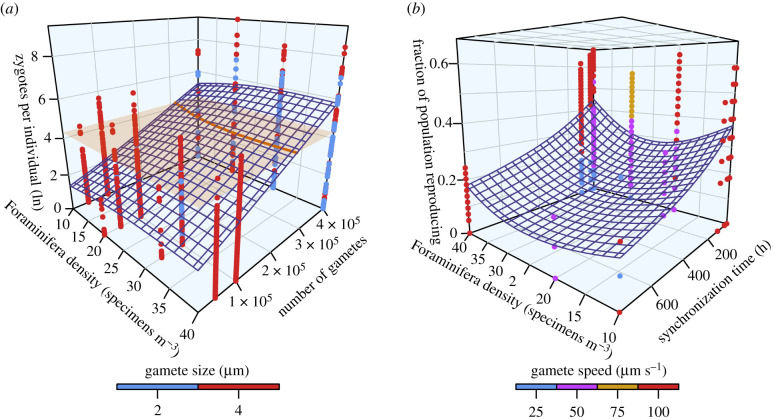
Reproductive success of planktonic Foraminifera depending on model parameters based on a GAM. (*a*) Number of zygotes produced per specimen across the population (log_e_-transformed). Orange horizontal plane and trace indicate 75 zygotes per foraminifer. (*b*) Fraction of the population successfully reproducing depending on model parameters.

The interpretation changes, however, when considering zygote production across the entire population (i.e. including unsuccessful individuals), which is more relevant for the retention of the population (table 3 and figure 2*a*). All modelled parameters significantly influenced reproductive success, with the success rate increasing with stricter synchronization of gamete release, increases in foraminiferal density, and number, size and speed of gametes. The CV of zygote production across the population has a mean of 4.35. The variation of reproductive success increased with a relaxation of the synchronization of gamete release but could be decreased by higher population densities and greater gamete speed, while size and number of gametes exhibited no controls on the variation of reproductive success across the population (electronic supplementary material, S2).

### 3.2. Proportion of the population reproducing

The reproductive success of the population is expressed as the fraction of individuals that were participating in at least one fusion of two gametes into a zygote. Within our model framework, 17.7% of the population reproduced successfully on average.

The population's reproductive success followed a zero-inflated beta distribution. The GAM indicates that the reproductive success was influenced by all tested parameters: Stronger synchronization, higher foraminifer densities, and larger, faster and more numerous gametes positively influenced reproductive success (table 3 and figure 2*b*; electronic supplementary material, S2). The CV of the population-wide success rate is 1.54 on average. The GAM on the gamma distribution shows an increase of variation with relaxed synchronization, lower foraminiferal density, and larger, slower gametes; the number of gametes exhibited no effect on success variation (table 3; electronic supplementary material, S2).

**Figure 3.**
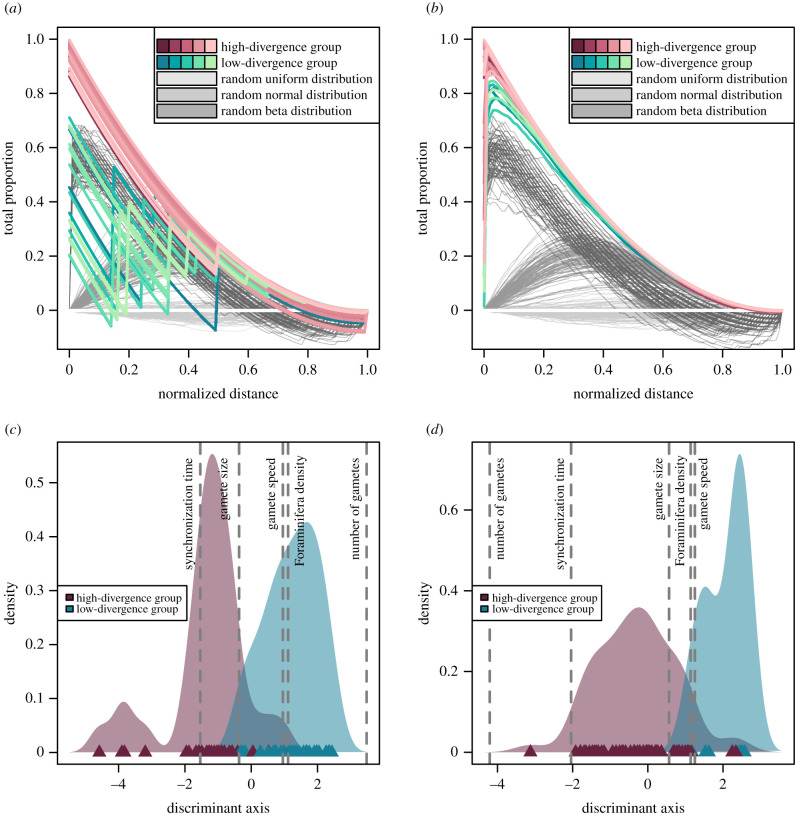
Divergence of reproductive success across individuals in planktonic Foraminifera under varying model set-ups. Ripley's *L* functions of number of reproductive events with another individual (*a*) and total number of zygotes produced per individual (*b*). The experiments were categorized into groups with higher and lower divergence between individuals, respectively: under low divergence, all individuals have comparable success; under high divergence, some individuals are very successful at the expense of others. Random examples (100 replications) of a uniform, normal and beta distribution are shown for comparison. Flexible discriminant analysis of the experiments, categorized according to Ripley's *L* functions, for number of reproductive partners (*c*) and number of zygotes produced (*d*). Data points are shown as triangles, with kernel density function as shaded area. Influence direction and weighting of different experimental parameters is indicated by grey, dashed lines.

Even in a scenario where a large proportion of the population reproduces successfully, it is possible that most zygotes are produced by only a few specimens. This can be tested by looking at the distribution of two parameters: (i) the number of successful reproductions of each individual with another foraminifer and (ii) the proportion of zygotes produced by each individual. Ripley's *L* function implies that the number of successful reproductions with different partners falls in either of two groups across our experiments (figure 3*a*,*c*). (i) Some experiments showed a low divergence following a shallow beta distribution or coming close to a normal distribution (electronic supplementary material, S2). Here, many individuals reproduced with a moderate number of different partners, but relatively few were either unsuccessful or reproduced with an extensive number of other individuals. Around half of the experiments (52%) fall in this group. (ii) In other experiments, the divergence followed a steep beta distribution, with most of the population reproducing with none or very few other individuals, while few individuals performed excessively well. An FDA shows that higher gamete production and population density promoted low divergence, while a relaxed synchronization time caused high divergence in the number of successful reproductions. For the number of produced zygotes per individual, all experiments followed a relatively steep beta distribution, but the divergence was lower in some experiments than in others (figure 3*b*,*d*; electronic supplementary material, S2). The low-divergence group (13% of all experiments) showed large numbers of individuals that did not produce any zygotes at all, but a rather uniform distribution of all successful individuals between very few and numerous zygotes produced. Within the high-divergence group, few individuals produced the vast majority of zygotes. A more even distribution was promoted by larger, faster gametes and a higher population density, while relaxed synchronization and a higher number of gametes bolstered a high divergence of the number of produced zygotes.

## 4. Discussion

### 4.1. Individual reproductive success and sustainability of the population

**Figure 4.**
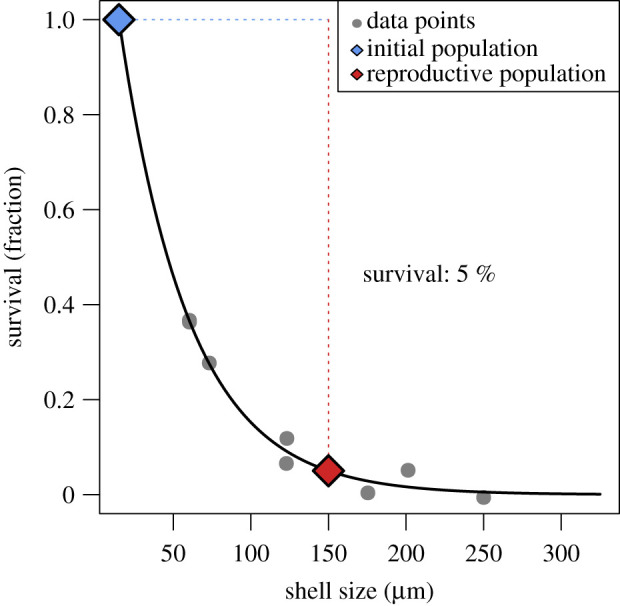
Estimated survival rate of planktonic Foraminifera from the zygote (initial population, 15 µm) to the reproductive stage (150 µm), based on plankton net and sediment data from Brummer *et al.* [52].

To evaluate the reproductive success rate necessary to maintain a population, data on the survival of zygotes in planktonic Foraminifera were needed. We estimated the probability of survival of planktonic foraminiferal zygotes to the reproductive stage using data from Brummer *et al.* [52, fig. 3], where foraminiferal abundances have been assessed over a wide shell size range. An exponential model for the survival of planktonic Foraminifera during ontogeny was fitted to these data (figure 4), assuming (i) a proloculus size (the initial foraminiferal shell, consisting of one chamber) of 15 µm [24,53] and (ii) an adult shell size of 125–150 µm [54]. We estimated the survival rate of planktonic Foraminifera from the zygote to the reproductive stage as *ca* 5%, which compares well with estimates for benthic Foraminifera (electronic supplementary material, S2) and survival curves in multicellular organisms [55,56]. Under this assumption, an average reproductive success of greater than 70–100 zygotes per individual foraminifer would suffice to sustain a viable population via sexual reproduction alone.

Only 9% of our experiments reached these numbers (compare electronic supplementary material, S3), which all showed stringent synchronization of gamete release within 12 h, large numbers (400 000) of gametes per specimen and high population densities (greater than or equal to 20 specimens m^−3^) (compare figure 2*a*). In nature, planktonic Foraminifera generally occur in very low abundances [30,31]. Temporal synchronization of their reproductive cycles was therefore suggested as a requirement to sustain a population, driven by either lunar cyclicity [7–9,32,37] or higher order cyclicities [9,57,58]. Our data support the need for temporally synchronized sexual reproduction to avoid unsustainably low numbers of gamete fusions. Additionally, a stronger synchronization reduces the variation in individual reproductive success (electronic supplementary material, S2), allowing to maintain stable population sizes. Triggers for gamete generation and synchronized release may include lunar tides or other physical oceanic parameters such as turbulence, turbidity or temperature [59].

Spatial concentration of adults could further aid reproductive efforts, for instance by sinking to a physical boundary layer like the halocline, which was repeatedly suggested for planktonic Foraminifera [9,58,60,61]. The turbulent nature of the ocean seems to promote the concentration of plankton into spatial clusters for reproductive purposes on smaller scales, although it may lead to segregation of populations on larger scales [31,62,63]. As we ran the models under laminar flow environments, this effect could not be simulated. However, the analyses by Borgnino *et al.* [63] suggest that turbulent flow would contribute to the spatial concentration process, by locally increasing population densities, which would help reaching the necessary population densities implied by our model. Spatial concentration combined with temporal synchronization of planktonic Foraminifera seems to be mandatory to sustain populations in a natural environment under the assumption of purely sexual reproduction (figure 2*a*). Chemical signalling between planktonic Foraminifera after concentration/pairing, which could trigger gamete production and has been observed in diatoms [64], could further enhance synchronization of gamete release.

The need for spatial or temporal synchronization could be relaxed if foraminiferal gametes possessed an active partner-detection mechanism. For some algae, a phototactic behaviour has been described [65], which ensures a uniform direction of movement of gametes to increase encounter rates. Chemotaxis could increase gamete encounter rates even more by enabling active partner tracking, if foraminiferal gametes overcame the sensitivity limit, e.g. by having sufficient receptors on their cell surface [66]. We decided against including such mechanisms in our models for lack of evidence but suggest concentrating empirical scientific research in this area in the future.

Our models suggest that populations would not be sustainable in species producing less than *ca* 250 000 gametes (figure 2*a*). In our estimates of gamete production rates, only the exceptionally large species *Trilobatus sacculifer* and *Orbulina universa* generate that many gametes. One possible solution may be that smaller species produce a greater number of smaller gametes (i.e. less than 4 µm) as opposed to fewer, larger gametes. In this case, the smaller gametes would likely have fewer energy reserves [34], reducing their survival time. However, this could be offset by the increased encounter rates generated by the increased gamete counts. Should this hypothesis be true, the equivalent of *r*- and *K*-strategists (enforced by adult shell size) may exist among planktonic Foraminifera, with potential implications for the adaptability of different species. Alternatively, if a strong spatial concentration or pairing of individuals took place prior to gamete release, this could levy the issue of low gamete counts by significantly raising encounter rates.

### 4.2. Distribution of reproductive success across the population

All models that would allow the largest fraction of the population (greater than 40%) to reproduce successfully are decidedly not the models that allow sustainable numbers of successful gamete fusions (electronic supplementary material, S3). The production of medium numbers of faster gametes seems to be most beneficial for an even divergence of success across the population (figure 2*b*; electronic supplementary material, S2). By contrast, the six models which led to sustainable populations had only 25% of the population successfully reproducing sexually on average. Except for a generally beneficial strict temporal synchronization, the model parameters which benefitted population-preservation through sexual reproduction were rather detrimental for the upkeep of gene flow and genetic intermixing within the population. This discrepancy does not seem to stem from the distribution of the number of reproductive events across individuals, as shown by Ripley's *L* (figure 3*a*; electronic supplementary material, S2). All models that sustained the population showed a low divergence of reproductive events, meaning that most individuals reproduced with a medium number of other individuals, thus keeping gene flow on a moderate level. Rather, it is the number of zygotes produced during these gamete cloud encounters that is skewed towards a large discrepancy between individuals. Each successful encounter of gamete clouds between two individuals can produce between one and hundreds of thousands of zygotes, depending on how many gametes of the two adult cells manage to fuse. In all models that can sustain the population, only few gamete cloud encounters produce large numbers of zygotes, so that most zygotes in the population are produced by few adult cells (figure 3*b*; electronic supplementary material, S2). Especially the high number of gametes necessary to sustain the population in our models increases the divergence in the number of zygotes individual Foraminifera produce (figure 3*d*).

Presumably, producing sufficient zygotes is more important to species survival than an even distribution of reproductive success across all individuals, although a combination of both would arguably be most beneficial. Accordingly, framework parameters maximizing zygote production would be favoured in terms of evolutionary fitness, even if it means sacrificing population-wide reproductive success and, thus, gene flow. If this held true, it could help explain some prior observations. (i) Norris [59] discussed in detail the problem of diversification and speciation in the open ocean plankton, which lives in an environment void of physical barriers which could prevent gene flow. Should planktonic Foraminifera indeed sacrifice gene flow for successful reproduction on the population level, it may help explain speciation between genetically isolated populations, even if they occur in sympatry [67–69] or undergo speciation in a homogeneous environment [29]. (ii) It could be shown that planktonic Foraminifera communities, when exposed to stressful environments, most often react with an adaptive response based on pre-existing variability rather than innovation through evolution [70–72]. The evolvability of planktonic Foraminifera may just be inherently low, but it may be that innovations simply cannot be fixated in the population due to reduced gene flow, which could, in turn, explain the spikes in variation sometimes observed in fringe environments [70].

### 4.3. Sexual versus asexual reproduction

Planktonic Foraminifera are the only major group of marine protists that have until recently been believed to reproduce nearly purely sexually through gametes [25]. While most protists are capable of sexual reproduction, asexual reproduction is frequently dominant [73]. This is even more intriguing when considering that planktonic Foraminifera descended from benthic Foraminifera, which exhibit alternating sexual and asexual reproductive cycles [20]. It was hypothesized that planktonic Foraminifera secondarily reduced their asexual reproductive cycle [29], as sexual reproduction is the more ancient reproductive mode [3,19]. This assumption, however, raises two main questions. (i) If asexual reproduction was secondarily reduced in all planktonic Foraminifera, this would have had to occur at least twice independently, since evidence suggests that modern planktonic Foraminifera are polyphyletic [28]. (ii) Sexual reproduction conveys several benefits, like the generation and spread of beneficial mutations [1,3] and the purging of harmful mutations [74]. Nevertheless, sexual reproduction bears a risk of failure since it depends on the encounter of the reproductive cells of two individuals. Planktonic Foraminifera would be an oddity in the protist world if they truly relied solely on sexual reproduction.

Recently, some studies documented the existence of asexual reproduction in planktonic Foraminifera [26,27]. The occurrence of asexual reproduction was so far estimated to comprise less than 1% of reproductive events [25], although other studies argue that it may even be the dominant mode of reproduction [75]. Davis *et al.* [27] showed that asexually produced offspring of planktonic Foraminifera are morphologically very variable, indicating a high phenotypic plasticity within the group. This phenotypic plasticity may under many circumstances be sufficient to ensure survival in variable environments even without sexual recombination and mutation. Asexual reproduction may therefore enable planktonic Foraminifera to sustain their populations with a reproductive mode that is more effective and less dependent on chance than sexual reproduction. It could ensure that even smaller species, which produce fewer gametes and would have difficulties surviving under our model when dependent on sexual reproduction alone, manage to endure. Coincidentally, asexual reproduction in planktonic Foraminifera has so far mainly been observed in smaller species in laboratory culture [25–27]. However, plasticity is not helpful when facing unprecedented environmental stress and decreases during evolutionary adaptive events [76]. We hypothesize that planktonic Foraminifera may have a more prevalent asexual reproductive cycle than previously believed that can ensure population sustenance. But they likely ensure evolutionary adaptation and gene flow through a temporarily and spatially synchronized sexual reproduction phase. Whether these phases alternate, as in many benthic Foraminifera, or are triggered by certain environmental conditions remains to be established. The fact that gametogenesis occurs regularly in laboratory culture may indicate that sexual reproduction is triggered by suboptimal environmental conditions, which would increase its value for adaptive evolution. This is consistent with evolutionary hypotheses indicating that in taxa capable of sexual and asexual reproduction, sexual reproduction is selectively favoured under scenarios of environmental adaptation, while asexual reproduction becomes rapidly dominant in stable environments with a stabilizing selection regime [77].

### 4.4. Open questions for future studies

Through these models, we identified critical questions as focus of future research to better understand reproduction of the marine protist plankton:

(i) How is synchronized gamete release triggered?
(ii) How/to what extent does spatial concentration of cells occur prior to sexual reproduction?
(iii) Do foraminiferal gametes have means for phototaxis or chemotaxis?
(iv) Are planktonic Foraminifera segregated into *K*-strategists and *r*-strategists?
(v) What is the relative contribution of sexual and asexual reproduction among planktonic Foraminifera and how does it vary across groups?

## Data accessibility

All model outcomes are available on PANGAEA in Weinkauf *et al.* [78]. Code for calculating gamete numbers is available on figshare in Weinkauf *et al.* [79].
